# Severe Neurological Sequelae and Radiological Findings in a Lost-to-Follow-Up Case of Hyperornithinemia-Hyperammonemia-Homocitrullinuria Syndrome

**DOI:** 10.7759/cureus.93690

**Published:** 2025-10-02

**Authors:** Ahmed Sarar Mohamed, Ahaan Gupta, Reem S Zakzouk

**Affiliations:** 1 Department of Medicine, Birmingham Heartlands Hospital, University Hospitals Birmingham NHS Foundation Trust, Birmingham, GBR; 2 Department of Radiology, Prince Sultan Military Medical City, Riyadh, SAU

**Keywords:** hyperornithinemia-hyperammonemia-homocitrullinemia syndrome, inborn errors of metabolism, mri brain, neuroradiology, pediatric neuroradiology, urea cycle disorders

## Abstract

Hyperornithinemia-hyperammonemia-homocitrullinuria (HHH) syndrome is a rare autosomal recessive urea cycle disorder caused by defective hepatic ornithine transport, leading to hyperammonemia and progressive neurological complications. We report the case of a patient who was treated for hyperammonemic crisis at birth and subsequently diagnosed with HHH syndrome. Management, including ammonia-lowering therapy and a low-protein diet, was initiated; however, due to significant socioeconomic barriers, he was lost to follow-up from the age of two. He re-presented at the age of 12 in a severely debilitated state with global developmental delay and refractory epilepsy. Investigations demonstrated radiological evidence of neurological damage, including corpus callosal atrophy, alongside biochemical and ultrasonographic features of hepatic dysfunction. This case highlights the critical importance of sustained treatment, multidisciplinary follow-up, and adequate social support in preventing irreversible complications of HHH syndrome.

## Introduction

Urea cycle disorders (UCDs) are rare inborn errors of metabolism caused by impaired hepatic conversion of ammonia to urea. Amongst these, the rarest is hyperornithinemia-hyperammonemia-homocitrullinuria (HHH) syndrome, an autosomal recessive disorder resulting from mutations in SLC25A15. This gene encodes the mitochondrial ornithine transporter 1 (ORNT1), and its dysfunction leads to failure of ornithine supply to ornithine transcarbamylase and subsequent hyperammonemia [1-3].

Diagnosis is suggested by the characteristic biochemical triad of hyperornithinemia, hyperammonemia, and homocitrullinuria, with confirmation by molecular testing [3]. The estimated incidence in the United States is less than one in 2,000,000 [4], and over 100 cases of this disorder have been described in the literature thus far, revealing a natural history associated with episodic hyperammonemic crises, characterised by acute neurologic deterioration, with symptoms such as lethargy, confusion, and coma occurring. In the long term, a plethora of complications may occur. These include cognitive impairment, developmental delay, and focal neurologic deficits [5].

Management focuses on maintaining metabolic stability, controlling clinical symptoms, and improving prognosis and long-term outcomes. This typically involves protein restriction, ammonia-scavenger therapy, and regular follow-up to prevent irreversible complications [5]. Despite these strategies, delayed diagnosis or interruption in care can have devastating outcomes.

Here, we present a case of neonatal-onset HHH syndrome in a patient lost to follow-up for a decade, resulting in severe clinical complications and interesting radiological findings.

## Case presentation

Initial presentations 

This male patient was born at full term via an uncomplicated vertex delivery but soon developed encephalopathic features, including lethargy, poor feeding, and hypotonia, alongside hyperammonemia, raising suspicion of a urea cycle disorder. Subsequent genetic testing revealed a defective SLC25A1 gene affected by the mutation c.658G>A, thus confirming the diagnosis of the rare HHH syndrome. 

He was subsequently followed up at the metabolic clinic of Prince Sultan Military Medical City, Riyadh, Saudi Arabia, where he was started on a low-protein diet and given ammonia-lowering therapy (L-arginine). He was asymptomatic until the age of two years, when he suffered a hyperammonemic crisis, which was preceded by admission to the hospital due to acute melena, where he was found to have a deranged coagulation profile secondary to HHH-related coagulopathy. He was given fresh frozen plasma (FFP) and vitamin K.

After discharge against medical advice, the patient neurologically deteriorated, with vomiting and irritability, and was subsequently readmitted. His workup revealed metabolic decompensation, with an elevated ammonia of 241 μmol/L (normal range for a two-year-old is <50 µmol/L), which peaked at 485 μmol/L in the subsequent 26-day hospital admission. Moreover, the peak international normalised ratio (INR) found was 6.2 (0.9-1.3). 

Following this episode of hyperammonemia secondary to GI bleeding, he was comprehensively examined and at this stage had no long-term complications, with motor, intellectual, and speech development being completely normal, and no seizures complicating his course. Furthermore, he was thriving well, with a weight of 10 kg, within normal limits for his age. Following this episode, the patient was not followed up at our centre for 10 years, and he did not take the required ammonia-lowering therapy, due to social issues, including geographic relocation and family instability. 

Complications after being lost to follow-up 

At the age of 12, he presented to the metabolic clinic in a markedly debilitated state, with global developmental delay, microcephaly, and intractable epilepsy being among the complications. His developmental delay was marked, and in the examination room, the patient was seen aimlessly wandering in the room with no clear speech, and his communication capacity included only two to three words.

His epilepsy was severely disabling and worsening, with a baseline of one to two seizures per day increasing to three times per day in the two weeks before presentation and occurring five times on the day before admission. In addition, the patient was severely underweight, with a weight of 27.8 kg and a height of 144 cm. Moreover, the patient had microcephaly, with a head circumference of 47 cm.

**Figure 1:**
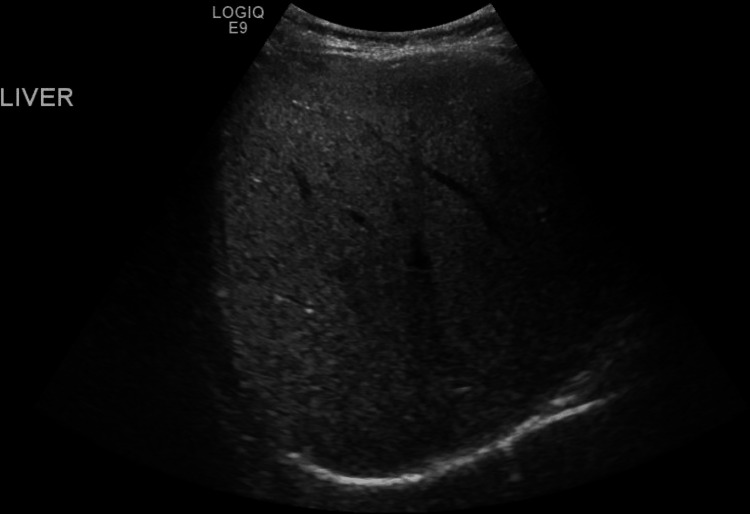
Longitudinal view of the right lobe of the liver, showing diffuse hyperechogenicity.

**Figure 2:**
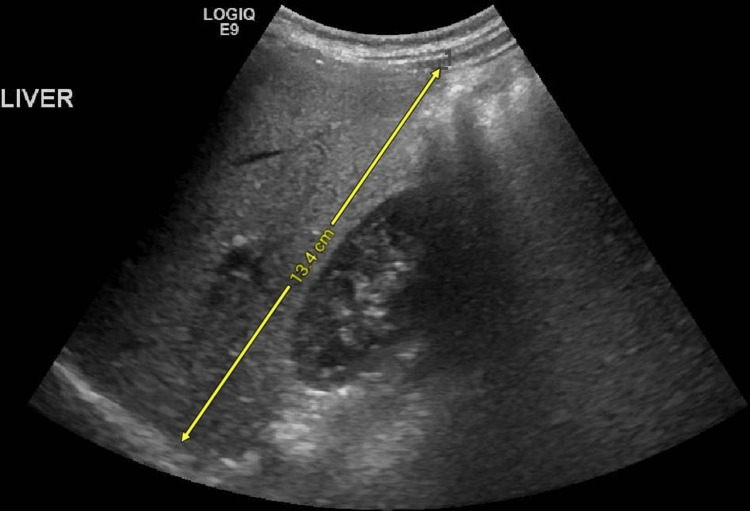
Longitudinal view of the right lobe of the liver, showing hepatomegaly with a craniocaudal length of 13.4 cm.

Our patient was thoroughly worked up with biochemical investigations, which revealed several metabolic and hepatic derangements (Table 1). The deranged liver function tests, together with the ultrasonographic findings of diffuse hepatic hyperechogenicity (Figure 1) and increased liver span of 13.4 cm (Figure 2), were consistent with UCD-induced liver damage.

**Table 1:** Laboratory investigations for our patient, including metabolic and liver function blood test results.

Lab tests	Patient blood level result	Normal range
Ornithine level	403 μmol/L	44 to 90 µmol/L
Ornithine to citrulline ratio	20	<10
Ammonia	94 μmol/L	11 to 32 µmol/L
Bilirubin	9.9 mg/dL	0.1-1.2 mg/dL
Alkaline phosphatase level	449 IU/L	44-147 IU/L

The EEG showed non-specific changes suggestive of encephalopathy, including frequent multifocal epileptiform discharges seen independently over bilateral hemispheres, more frequently over the posterior right head region. 

**Figure 3:**
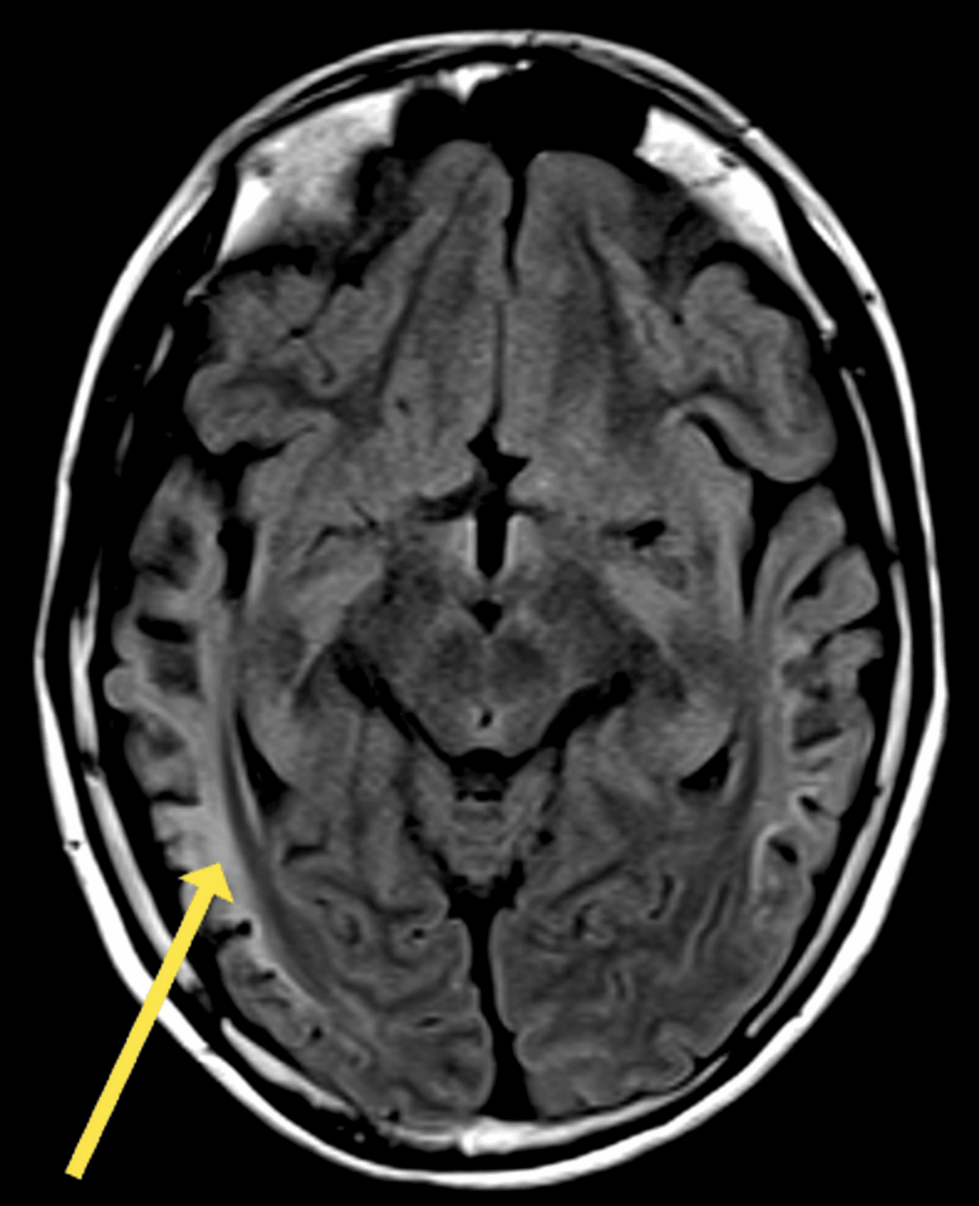
Axial fluid attenuated inversion recovery (FLAIR) magnetic resonance image revealing atrophic and gliotic changes; arrow indicates peripheral white matter hyperintensity (arrow).

**Figure 4:**
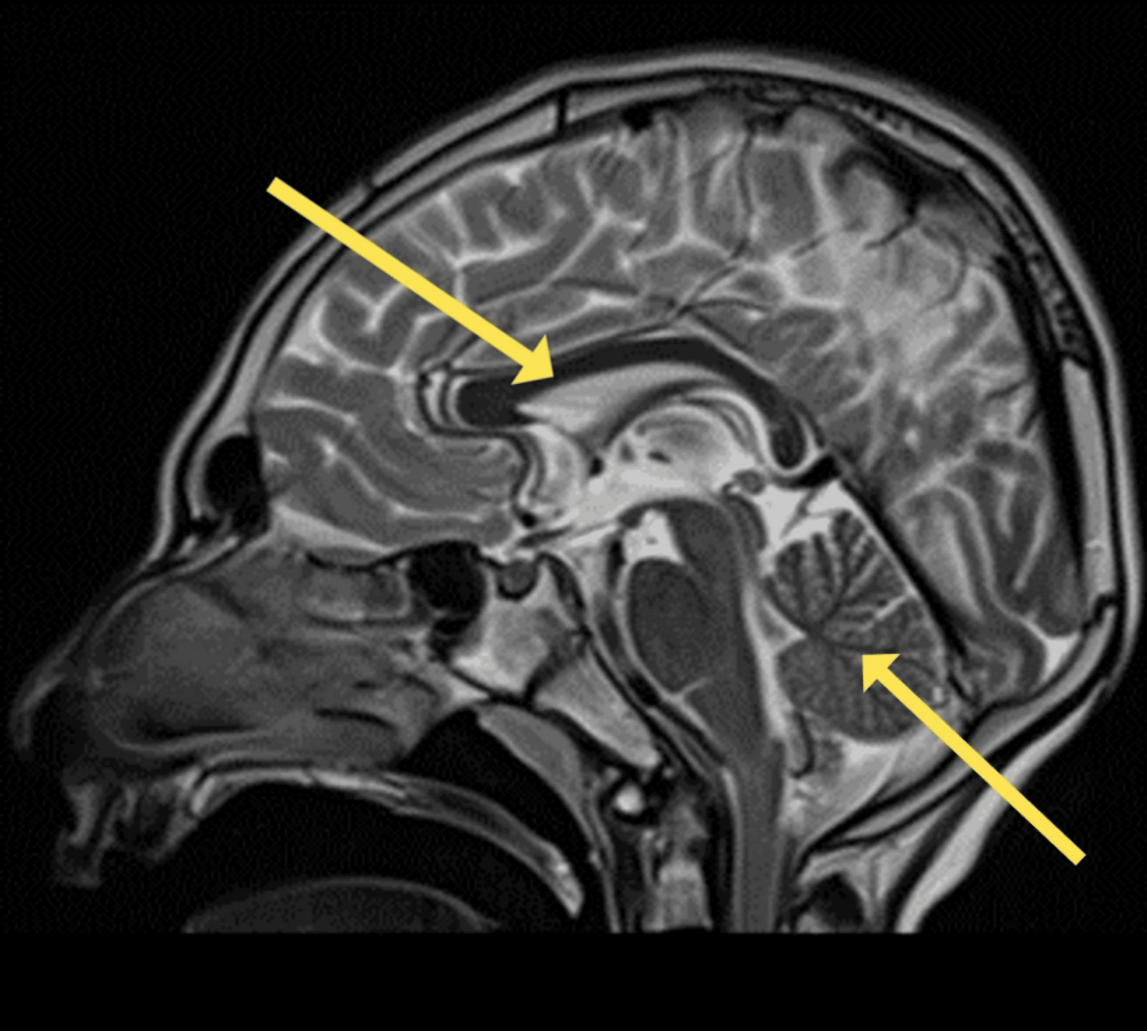
Sagittal T2-weighted magnetic resonance image revealing microcephaly and diffuse atrophy of the corpus callosum and cerebellum (arrows)

Magnetic resonance imaging (MRI) demonstrated widespread cortical atrophy with relative occipital sparing, accompanied by gliotic changes in the white matter, evident as peripheral hyperintensities. Sagittal T2-weighted images further revealed microcephaly with diffuse atrophy of the corpus callosum and cerebellum (Figures 3-4).

## Discussion

This case demonstrates the importance of management and regular follow-up in patients with UCD, as the failure to do so leads to the accumulation of brain damage secondary to hyperammonemia, through cerebral oedema that occurs due to the osmotic effects of the consequent build-up of glutamine [6]. As a result, a plethora of neurological complications may ensue, as shown in this case. The most pertinent complications in our patient, namely intellectual disability and seizures, are relatively common in HHH patients, afflicting 68% and 35% of patients, respectively [5]. Although behavioural disorders and progressive motor complications such as weakness, spasticity, and cerebellar ataxia are common complications [5], this was not found in our patient. Additionally, the ornithine level of 403 in our patient was on the lower side of the reported range of 216 to 1915 μmol/L [5] despite our patient being untreated. 

Our case was neonatal in onset, which contrasts with the majority of cases [7], and although two hyperammonemic crises were suffered during infancy, the expected neurocognitive complications were later in onset. Also consistent with the literature [7] was the liver dysfunction manifested in lab and ultrasonographic findings. Although mild coagulopathy and liver dysfunction do commonly occur in HHH [5,7], in our patient, this presented in a more sinister way, with a GI bleed and a markedly elevated INR, precipitating a hyperammonemic crisis at the age of two years. Although a hyperammonemic crisis in HHH precipitated by a GI bleed has previously been described in a case study [8], the bleeding in that case was secondary to a known cause of peptic ulcer disease. In our patient, there was no underlying GI pathology found that could have caused the bleeding. In addition, the coincidence of elevated INR and neonatal hyperammonemia in HHH has been previously described [1]; however, only a mild elevation of 2.3 was reported, and no bleeding secondary to the coagulopathy was reported. 

Neurological damage also manifested radiologically, with findings of bilateral peripheral and subcortical white matter hyperintensity consistent with gliosis, a reactive proliferation and hypertrophy of astrocytes secondary to brain injury, eventually resulting in the formation of scar tissue [9]. Other neuroradiological findings of chronic brain damage included bilateral cortical atrophy. Although the atrophy was extensive, the occipital lobe was relatively spared, in keeping with the general literature on urea cycle disorders [10-12]. 

Additionally, our patient had corpus callosum atrophy on brain MRI, a manifestation that likely occurs due to axonal degeneration, with a resultant decrease in the metabolic activity of the cerebral cortex [13]. The coincidence of corpus callosal atrophy with speech and intellectual delay in our patient is consistent with multiple studies that correlate atrophy of the corpus callosum with cognitive deficits in neurodegenerative pathology [13-15]. Neurocognitive deficit is also associated with congenital defects in the corpus callosum [16] as well as secondary pathology of the corpus callosum, such as in multiple sclerosis [17] or infarction [18,19].

## Conclusions

This case report signifies that HHH syndrome, although a rare condition, can lead to severe complications in patients due to metabolic disruptions. Our patient experienced a range of complications that impacted his quality of life, including metabolic crisis, developmental delay, neurological deficit and cognitive impairment. Although diagnosed at an early age, our patient was lost to follow-up due to social issues and developed long-term complications. This signifies the importance of early treatment and close monitoring with regular follow-up to avoid long-term damage. Family members of the patient should be educated on identifying signs of metabolic crises and when to seek urgent medical attention. We recommend that further research is warranted to improve management and follow-up requirements. Affected individuals should be offered long-term monitoring and support to improve their health outcomes and overall quality of life.
